# Characterization of intestinal fibrosis in cats with chronic inflammatory enteropathy

**DOI:** 10.1111/jvim.16688

**Published:** 2023-04-13

**Authors:** Yuvani Bandara, Simon L. Priestnall, Yu‐Mei Chang, Aarti Kathrani

**Affiliations:** ^1^ Royal Veterinary College University of London London UK

**Keywords:** albumin, cobalamin, immunohistochemistry, inflammatory bowel disease, outcome, prognosis, weight

## Abstract

**Background:**

Intestinal fibrosis (IF) is commonly identified on histopathology of intestinal biopsy specimens (IBSp) from cats with chronic inflammatory enteropathy (CIE) however, its clinical relevance is unknown.

**Objectives:**

Characterize and determine the clinical relevance of IF in cats with CIE.

**Animals:**

Sixty‐five client‐owned cats diagnosed with CIE after gastrointestinal histopathology from a single referral hospital in the United Kingdom.

**Methods:**

Medical records were retrospectively searched for cases of CIE on the basis of histopathology of IBSp. The IBSp from eligible cats were re‐reviewed by a single board‐certified veterinary pathologist for inclusion. Masson's trichrome (MT) stain and immunohistochemical labeling using antivimentin and anticollagen I antibodies to identify IF. For each case, various variables at the time of diagnostic investigation were recorded and referring veterinarians were contacted for follow‐up information.

**Results:**

Mucosal fibrosis was identified in 51% of duodenal and 76% of colonic hematoxylin and eosin (HE)‐stained IBSp. Vimentin labeling and MT staining identified additional cases of IF in 65% and 58% of the duodenal biopsy specimens, respectively. Vimentin labeling detected IF in 79% of the colonic biopsy specimens. Positive vimentin labeling and MT staining of the colonic mucosa were associated with decreased likelihood of attaining clinical remission and increased risk of death because of CIE (*P* < .05).

**Conclusions and Clinical Importance:**

Additional stains at initial histopathologic examination of IBSp allow for better identification of IF compared to routine HE staining. Identification of IF in colonic biopsy specimens by vimentin immunolabeling and MT staining may provide prognostic information in cats with CIE.

**Abbreviations**
ACA: anticollagen I antibody
ALP: alkaline phosphatase
ALT: alanine aminotransferase
BCS: body condition score
BW: body weight
CCS: composite collagen score
CE: chronic enteropathy
CI: confidence interval
CIE: chronic inflammatory enteropathy
ECM: extracellular matrix
ER: epitope retrieval
FFPE: formalin‐fixed paraffin embedded
fPLI: feline pancreatic lipase immunoreactivity
FT: full‐thickness
GI: gastrointestinal
HE: hematoxylin and eosin
IBD: inflammatory bowel disease
IBSp: intestinal biopsy specimens
IF: intestinal fibrosis
IHC: immunohistochemistry
iP: inorganic phosphorous
MT: Masson's trichrome
OR: odds ratio
SCL: small cell lymphoma
SI: small intestinal
TLI: trypsin‐like immunoreactivity
TP: total protein
TT4: total thyroxine
WSAVA: World Small Animal Veterinary Association

## 1 INTRODUCTION

Chronic inflammatory enteropathy (CIE) in cats describes a group of idiopathic diseases resulting in gastrointestinal (GI) signs of at least 3 weeks' duration. Definitive diagnosis requires ruling out all known causes of chronic GI signs, including infectious, obstructive, neoplastic and extraintestinal diseases by histopathological examination.^1^ The exact etiology of CIE in cats is currently unknown, but its hypothesized etiopathogenesis is extrapolated from inflammatory bowel disease (IBD) in humans, another similar enteropathy. Consequently, CIE in cats is postulated to occur because of genetic defects in the ability to differentiate between commensal vs pathogenic bacteria, aberrant innate immune system responses, and elusive environmental factors in susceptible hosts.^1^, ^2^, ^3^

The presence of intestinal mucosal fibrosis is commonly noted on histopathologic examination of intestinal biopsy specimens (IBSp) of cats with CIE.^1^, ^4^ Intestinal fibrosis (IF) is characterized by an alteration in collagen metabolism after chronic inflammation, resulting in its excessive and irreversible deposition in the extracellular matrix (ECM).^5^ An increase in the resident mesenchymal cell population responsible for the deposition of collagen is the principal mechanism in intestinal fibrogenesis.^6^ Mesenchymal cells can be broadly classified into fibroblasts, myofibroblasts, and smooth muscle cells. Despite IF occurring in cats with CIE, its clinical relevance remains unknown.

In humans with IBD, IF is an expected complication and a distinct cause of patient morbidity and mortality.^5^, ^7^, ^8^, ^9^ In humans with IBD, myofibroblasts have been identified as key cellular mediators of collagen deposition, with collagen types I and III considered the 2 major ECM proteins associated with IF.^10^, ^11^, ^12^ Intestinal fibrosis persists in a self‐perpetuating manner in the absence of inflammation^13^ with evidence also indicating that ECM stiffness itself is capable of perpetuating collagen deposition.^14^ Therefore, targeting signaling pathways that lead to upregulation of IF in humans with IBD is a diagnostic and therapeutic focus.^15^, ^16^

Currently, identification and quantification of IF in cats with CIE are based solely on the assessment of morphologic features using World Small Animal Veterinary Association (WSAVA) histopathologic scoring of hematoxylin and eosin (HE)‐stained tissue sections. However, concern exists among veterinary pathologists that IF may be underreported based on HE staining alone and, additionally, may be masked by edema or missed because of sample orientation.^17^ Therefore, use of additional stains, such as Masson's trichrome (MT) or immunolabeling for vimentin and collagen I, may better identify the presence, pattern and pathology of mucosal fibrosis.

Our aim was to determine the frequency of IF in cats with CIE by utilizing additional stains and immunolabeling. We also aimed to correlate the presence of IF with clinical findings, results from diagnostic investigations, and outcome to further characterize its clinical relevance.

## 2 MATERIALS AND METHODS

### 2.1 Study cats

A single university referral hospital's Feline Chronic Enteropathy (CE) Archive, Feline GI Biopsy Archive and medical records database were retrospectively searched by a single operator for cats with a diagnosis of CIE after GI histopathology between June 2008 and November 2021. For each case retrieved, the electronic clinical records were reviewed and information gathered pertaining to each cat's referral visit at the time of GI biopsy specimen retrieval. Inclusion criteria consisted of: (1) a minimum of 3 weeks of persistent or intermittent GI signs (vomiting, diarrhea, hyporexia, and weight loss); (2) known patient signalment (breed, age, sex, and neuter status), body weight (BW) and body condition score (BCS) assessed on a 9‐point scale^18^; (3) diagnostic investigations including: CBC, serum biochemistry profile, serum cobalamin or folate concentrations or both, serum total thyroxine (TT4) concentration if >8 years of age,^19^ pancreatic function testing (feline pancreatic lipase [fPLI] and trypsin‐like immunoreactivity [TLI]) as indicated by historical findings and physical examination findings, fecal parasitology with or without empirical deworming, *Tritrichomonas foetus* PCR assay if large intestinal diarrhea was present and complete transabdominal ultrasound examination; (4) histopathologic examination by a board‐certified veterinary pathologist of IBSp obtained by laparotomy or endoscopy to allow a final diagnosis of CIE; (5) information on treatment prescribed at discharge from the referral hospital categorized as: dietary, antibiotic, antibiotic and dietary, immunosuppressive, or immunosuppressive and dietary.

### 2.2 Follow‐up information

A minimum of 6 months' follow‐up was required for all cats. An exception was cats that died or were euthanized for any cause before 6 months after IBSp retrieval. Follow‐up information was gathered by telephone contact or email questionnaire from the referring veterinarian who originally referred the cat. Follow‐up information included: whether the cat was in clinical remission, its current treatment regimen, patient outcome (alive or dead and reason for death), and the date of this information. Clinical remission was defined as the absence of GI signs (vomiting, diarrhea, hyporexia, weight loss). Follow‐up time was defined as the time from histopathologic diagnosis of CIE to the date of last consultation with the referring veterinarian as stated in the questionnaire.

### 2.3 Initial histopathologic assessment

For cats that met inclusion criteria, original formalin‐fixed paraffin embedded (FFPE) and HE‐stained slides of duodenal and colonic biopsy specimens were reviewed again by a single board‐certified veterinary pathologist, with a special interest in GI disease, to confirm the diagnosis of CIE and were assigned a WSAVA histopathology score.^20^ Absence of intestinal mucosal fibrosis was defined as a narrow band of stroma up to 1 to 2 fibroblasts in width, whereas the presence of intestinal mucosal fibrosis was described as crypts separated by a narrow band of stroma with a width of >2 fibroblasts.^20^ Cats were excluded if the HE‐stained IBSp were of poor quality, poor tissue orientation or absent. Biopsy specimens suspicious for alimentary neoplasia on examination underwent further immunohistochemical labeling using CD3 and CD20 antibodies. Intestinal biopsy specimens that were confirmed as alimentary neoplasia on immunohistochemistry (IHC) were excluded.

### 2.4 Masson's trichrome staining and IHC for vimentin

Staining was performed using Masson's trichrome (MT) and IHC for vimentin on 4‐μm thick FFPE tissue sections.

For immunolabeling, primary antibodies and retrieval conditions were as follows: mouse monoclonal anti‐vimentin antibody (1:500, Agilent, Stockport, England); pH 6.0 buffer (epitope retrieval solution 1 [ER1], Leica Biosystems) for 10 minutes on a Leica BondMax Autostainer using the Bond Polymer Refine detection kit (DS9800, Leica Biosystems) which includes a peroxide block, post‐primary, polymer reagent, 3‐3′‐diaminobenzidine chromogen, and hematoxylin counterstain in a ready‐to‐use format. Internal positive tissue controls for vimentin (blood vessels) were available on each section of feline intestine.

### 2.5 Evaluation of MT staining and immunolabeling of vimentin

Analysis of MT‐stained and immunolabeled IBSp was performed by a single board‐certified veterinary pathologist, at magnification ×100, over 5 nonoverlapping fields within the mucosa. Fields were selected based on regions with the highest staining intensity or immunolabeling intensity.

**FIGURE 1:**
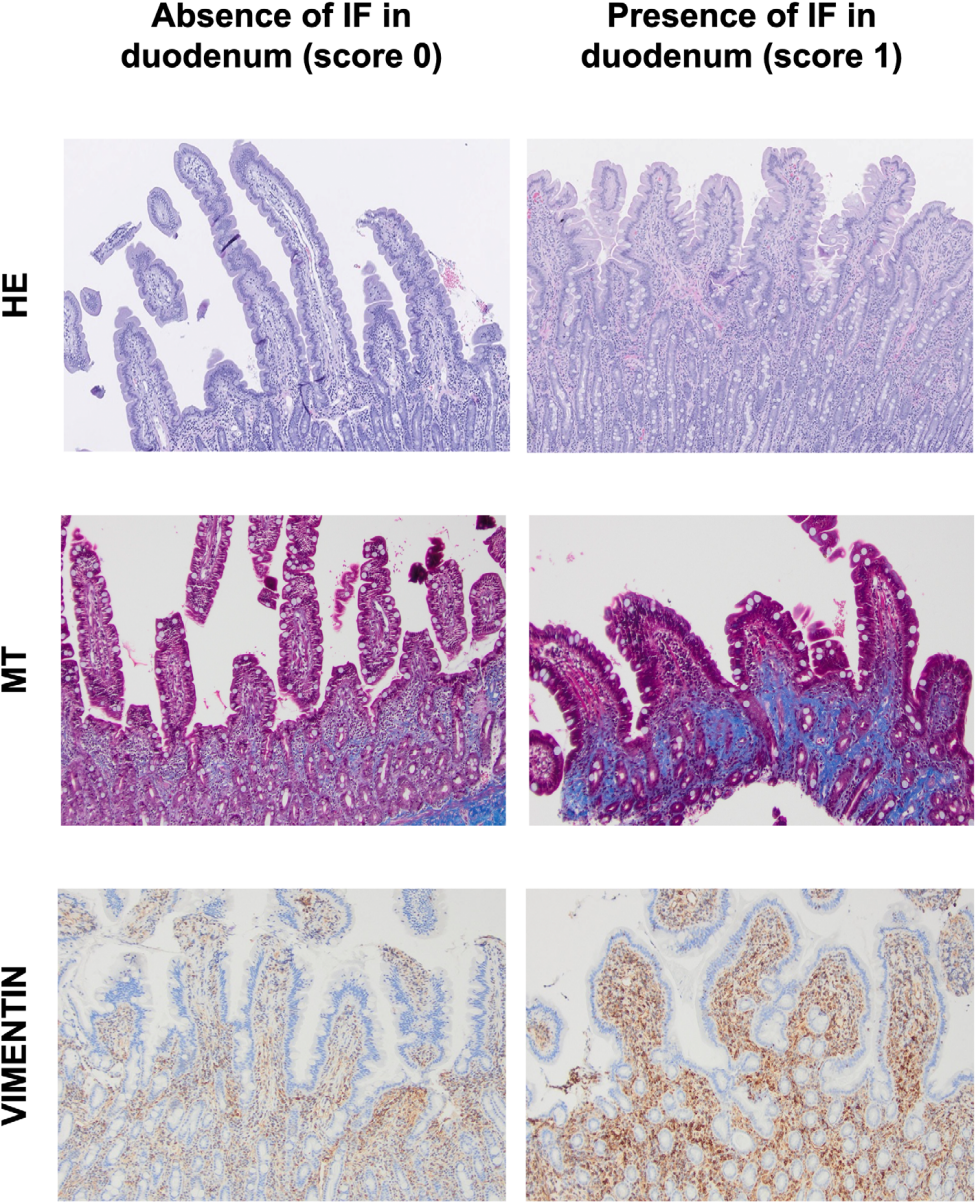
Photoguide for the assessment of the presence of mucosal fibrosis in duodenal biopsy specimens (×100) from cats with CIE having undergone routine staining with HE and additional staining with MT and IHC for vimentin. Normal submucosal collagen is visible in the duodenal biopsy specimen stained with MT, middle left.

**FIGURE 2:**
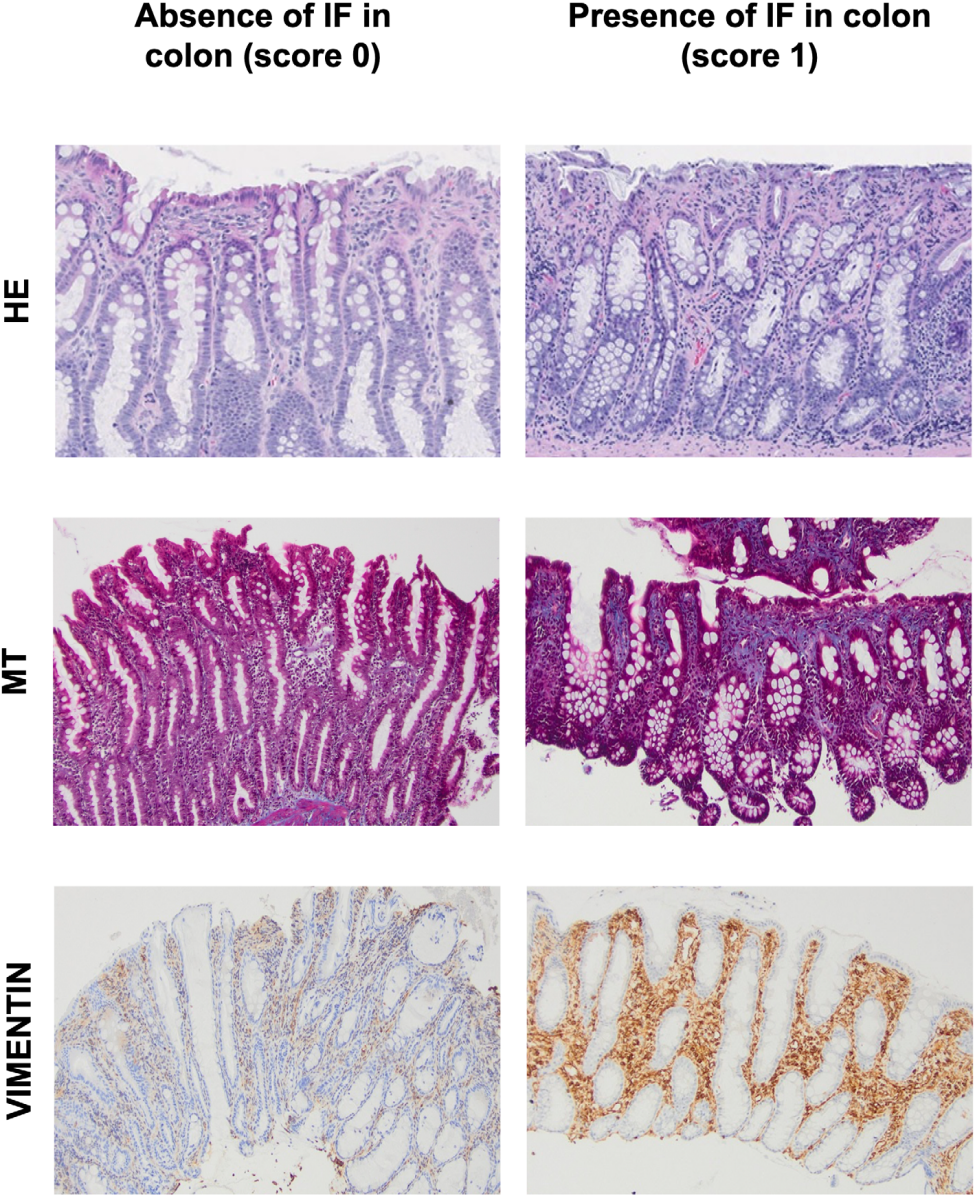
Photoguide for the assessment of the presence of mucosal fibrosis in colonic biopsy specimens (×100) from cats with CIE having undergone routine staining with HE and additional staining with MT and IHC for vimentin.

Staining with MT was visually graded as 0: no stain uptake and 1: positive stain uptake. Normal feline intestine, which contained MT staining of the submucosa and serosa, was used as a positive control. Considering that the normal feline intestine contains expected vimentin‐positive mesenchymal components, notably a resident population of lymphocytes and plasma cells, immunolabeling with vimentin was based on all other mesenchymal components such as fibrocytes, fibroblasts, smooth muscle, and blood vessels after exclusion of inflammatory cells. Immunolabeling for vimentin was scored as 0: absent labeling (inflammatory cells only) and 1: positive labeling. Photo guides, visually detailing how slides were scored, are presented as Figure 1 for duodenal specimens and Figure 2 for colonic specimens.

### 2.6 Immunohistochemistry for collagen I

Twenty duodenal biopsy specimens, with an individual WSAVA duodenal mucosal fibrosis score of ≥1 as scored during grading for the current study, were selected for further immunolabeling to determine the presence of collagen I. Labeling was performed using a recombinant rabbit monoclonal anticollagen antibody (ACA; Abcam PLC Recombinant Anti‐Collagen I antibody [EPR7785] [ab138492], Cambridge, England). Retrieval conditions for collagen I were as follows: 1:500, pH 9.0 buffer (epitope retrieval solution 2, Leica Biosystems) for 10 minutes. Immunohistochemistry was performed on the Leica BondMax Autostainer using the Bond Polymer Refine detection kit (DS9800, Leica Biosystems). Internal positive tissue controls (submucosal collagen) were available on each section from feline intestine.

### 2.7 Evaluation of immunolabeling of collagen I

Collagen I deposition in the duodenal mucosa was graded by visually assessing its (1) labeling intensity and (2) frequency, over 5 nonoverlapping (×100) fields, to give a composite collagen score (CCS) which was calculated as a multiple of these 2 values.^21^ Regions with the highest immunolabeling intensity were selected.

**FIGURE 3:**
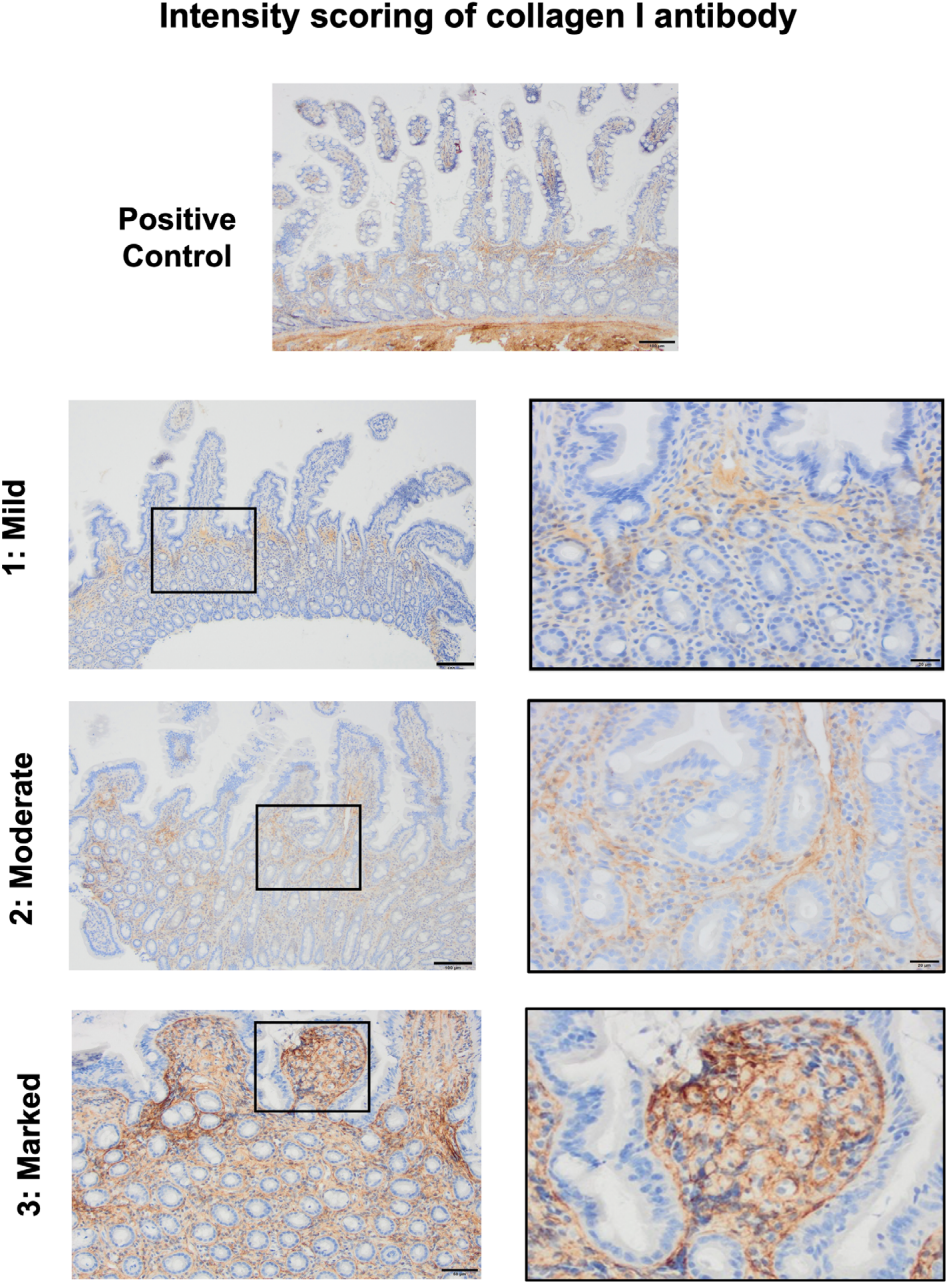
Photoguide for the assessment of the intensity of mucosal collagen labeling in duodenal biopsy specimens (×200) from cats with CIE having undergone IHC using a collagen I antibody. Positive control image depicts collagen labeling of the duodenal submucosa. Black squares indicate areas that appear magnified in the right column.

Labeling intensity, with collagen I in the duodenal mucosa, was graded as 0: absent, 1: mild, 2: moderate or 3: marked. Frequency was graded as a visual estimate of the percentage of area labeled as 0: <25%, 1: 25% to 50%, 2: 51% to 75%, or 3: 76% to 100%. The CCS, therefore, was graded with scores of 0 to 1: mild collagen labeling, 2 to 4: moderate collagen labeling and ≥6: marked collagen labeling. A photo guide, visually detailing how collagen I labeling intensity was scored, is presented as Figure 3. Because collagen I frequency was graded over multiple fields and tissue sections, only labeling intensity, and not frequency, is represented graphically.

### 2.8 Statistical analyses

All statistical analyses were performed using the software IBM SPSS Statistics (Version 28.0.0.0).

Continuous numerical variables were summarized as a median value (minimum and maximum) and categorical variables were summarized as frequencies (proportion).

The clinicopathologic variables included in the analysis consisted of: BW; BCS; history of diarrhea or weight loss (yes vs no); serum total protein (TP), albumin, inorganic phosphorus (iP), and cobalamin concentrations; alanine aminotransferase (ALT) and alkaline phosphatase (ALP) activities; thickening of intestinal muscularis propria layer on transabdominal ultrasound examination (yes vs no), and composite WSAVA score of the duodenum or colon. Attainment of clinical remission was categorized as yes vs no. Outcome was categorized as cats that died because of GI disease at the time of follow‐up vs cats that died of other causes and cats that were alive at the time of follow‐up. The presence of IF (dependent variable) was categorized as: MT stain (yes vs no), vimentin labeling (yes vs no) and collagen I deposition using the CCS.

Univariable binary logistic regression was performed to assess the association between clinicopathologic variables and the presence of IF using MT stain and immunolabeling for vimentin. Results were presented as odds ratios (OR) and 95% confidence intervals (CI). Because of an extreme categorical problem (zero in contingency table), Kendall's tau‐b was employed to assess the correlation between the presence of IF in colonic biopsy specimens and the following variables: history of diarrhea and thickening of muscularis propria layer on ultrasound examination, and these results were reported descriptively. Kendall's tau‐b was used to examine the association between IF and the attainment of clinical remission and outcome of death because of GI disease.

For the 20 duodenal biopsy specimens that underwent IHC for ACA, a Spearman's rank correlation (*r*_s_) was used to evaluate the correlation between CCS and all continuous clinicopathologic variables. Kendall's tau‐c correlation was reported between CCS and ordinal/binary variables.

Type I error rate for all statistical analyses was set at 0.05.

## 3 RESULTS

### 3.1 Study cats

Eighty cats met inclusion criteria. On histopathologic examination of the HE‐stained slides by the board‐certified veterinary pathologist, 6 cats (8%) were suspicious for alimentary lymphoma. Further immunohistochemical labeling for CD3 and CD20 confirmed T‐cell lymphoma and epitheliotropic T‐cell lymphoma in 2 cats and 1 cat, respectively. An additional 12 cats were excluded because of poor sample quality or orientation. Therefore, 15 cats (19%) in total were excluded from all further analyses.

The remaining 65 cats were eligible to enter the study with a diagnosis of CIE made on HE‐stained slides between September 2011 and August 2021. Demographic characteristics and clinical signs of the study group are presented in Table 1.

**TABLE 1:** Demographic data of the 65 cats that met final study inclusion criteria.

Number of cats in study	65
*Demographic information*	
Median age in months (min‐max)	96 (10‐206)
Median body weight in kilograms (min‐max)	4 (1.69‐9.3)
Median body condition score (min‐max)	4 (1‐8)
Sex	33 male neutered, 1 male entire, 31 female neutered
Breeds	38 Domestic Shorthair, 4 Bengal, 4 Siamese, 4 Domestic Longhair, 3 Burmese, 3 Maine Coon, 2 Exotic Shorthair, 2 Oriental Shorthair, 1 British Longhair, 1 British Shorthair, 1 Cornish Rex, 1 Persian, 1 Ragdoll
*Clinical signs*	
Median duration (min‐max)	16 weeks (2‐216)
Presentation of clinical signs	Vomiting (36/65 cats, 55%) Weight loss (33/65 cats, 51%) Diarrhea (32/65 cats, 49%) Hyporexia (22/65 cats, 39%) Presentation of 1 clinical sign (26/65 cats, 40%) Combination of 2 clinical signs (24/65 cats, 37%) Combination of 3 clinical signs (11/65 cats, 17%) Combination of 4 clinical signs (4/65 cats, 6%)

### 3.2 Ultrasound examination and intestinal biopsy specimen collection

All 65 cats in the study underwent transabdominal ultrasound examination. Of these 65 cats, 27 (42%) were reported to have thickening of the small intestinal (SI) muscularis layer. Intestinal thickening was described as diffuse in 10 cats (37%). Areas of localized intestinal thickening were described in the duodenum only in 1 cat (4%), of the jejunum only in 6 cats (22%), of the jejunum and ileum in 4 cats (15%), of the ileum only in 1 cat (4%), and of the colon only in 5 cats (18%).

Full‐thickness (FT) biopsy specimens were obtained by laparotomy in 9 cats (14%) and partial‐thickness biopsy specimens were obtained by endoscopy in 56 cats (86%). Partial‐thickness and FT biopsy specimens of the duodenum were collected in 50 cats (77%) and 7 cats (11%), respectively. In 2 cats (3%), FT ileal and jejunal biopsy specimens also were collected. Partial‐thickness biopsy specimens of the colon were collected in 29 cats (47%). Partial‐thickness biopsy specimens of both the duodenum and colon were collected in 23 cats (35%).

### 3.3 Treatment

At discharge from the university referral hospital, 37 cats (57%) were prescribed treatment consisting solely of dietary management. The diet consisted of a limited‐ingredient novel protein‐based diet in 16 cats (43%) or a commercial therapeutic hydrolyzed diet in 21 cats (57%). Six cats (9%) were prescribed antibiotics (metronidazole [n = 3], metronidazole and pradofloxacin [n = 1], marbofloxacin [n = 1] or amoxicillin‐clavulanic acid [n = 1]) in addition to dietary therapy. Four cats (6%) were prescribed immunosuppressive treatment only (prednisolone [n = 3] or prednisolone and chlorambucil [n = 1]). Eighteen cats (28%) received an immunosuppressive medication (prednisolone [n = 12], prednisolone and chlorambucil [n = 5], or cyclosporine [n = 1]) along with dietary therapy.

### 3.4 Questionnaire response and outcome information

Questionnaire response rate by referring veterinarians was 83% (n = 54) of the 65 cats in the study group. For the 11 cats (17%) for which no follow‐up information was obtained, the reason was that the medical records were unavailable (n = 6) or these cats were lost to follow‐up (n = 5).

Of the 54 cats for which follow‐up information was obtained, 23 cats (43%) were alive at the time of follow‐up (median, 916 days; range, 183‐2113 days after histopathologic diagnosis) and 11 cats (20%) died because of other causes (median, 323 days; range, 24‐2580 days after histopathologic diagnosis). The remaining 20 cats (37%) were euthanized because of GI disease within a median of 129.5 days after histopathologic diagnosis (range, 8‐2970 days).

### 3.5 Identifying IF in duodenal and colonic biopsy specimens with the addition of MT staining and IHC for vimentin

Of the 57 HE‐stained duodenal biopsy specimens, WSAVA scoring by the board‐certified veterinary pathologist found no evidence of mucosal fibrosis in 28 specimens (49%), mild mucosal fibrosis in 27 specimens (47%), and moderate mucosal fibrosis in 2 specimens (4%). Of the 29 HE‐stained colonic biopsy specimens, WSAVA scoring by the board‐certified veterinary pathologist found no evidence of mucosal fibrosis in 7 specimens (24%) and mild mucosal fibrosis in 22 specimens (76%).

The use of MT staining and IHC labeling for vimentin identified additional cases of IF in 33 (58%) and 37 (65%) of the 57 duodenal biopsy specimens, respectively. An increase in cases of IF also was noted after vimentin labeling of the colon, with IF detected in 23 (79%) of the 29 colonic biopsy specimens. Further information regarding detection of IF in the duodenal and colonic biopsy specimens, as detected using MT staining and immunohistochemical labeling for vimentin, is presented in Table 2.

**TABLE 2:** Presence of IF in 57 duodenal and 29 colonic biopsy specimens using hematoxylin and eosin stain, Masson's trichome stain and immunolabeling of vimentin.

Specimen	Number of specimens	Hematoxylin and eosin		Masson's trichrome		Vimentin	
		Positive	Absent	Positive (score 1)	Absent (score 0)	Positive (score 1)	Absent (score 0)
Duodenum	57	29 (51%)	28 (49%)	33 (58%)	24 (42%)	37 (65%)	20 (35%)
Colon	29	22 (76%)	7 (24%)	21 (72%)	8 (28%)	23 (79%)	6 (21%)

### 3.6 Results of association analyses for MT staining and IHC of vimentin

Univariable logistic regression found a positive relationship between BW (OR, 1.83; 95% CI, 1.05‐3.18; *P* = .03) and BCS (OR, 1.52; 95% CI, 1.52‐2.29; *P* = .04) and vimentin labeling of duodenal biopsy specimens.

Kendall's tau‐b indicated that a history of diarrhea showed a weak positive correlation with MT staining on colonic biopsy specimens (ꞇ 0.152, *P* = .04) but no similar correlation was identified with vimentin immunolabeling (ꞇ 0.204, *P* = .06). Kendall's tau‐b also identified that thickening of the intestinal muscularis propria layer on ultrasound examination was positively correlated with vimentin labeling on colonic biopsy specimens (ꞇ 0.342, *P* = .003). Univariable logistic regression showed no relationship between thickening of the intestinal muscularis propria layer on ultrasound examination and MT staining on colonic biopsy specimens (OR, 0.13; 95% CI, 0.01‐1.25; *P* = .08). Increased vimentin labeling and MT staining of colonic biopsy specimens were both significantly correlated with failure of attaining clinical remission (ꞇ −0.335, *P* = .03). Increased vimentin labeling (ꞇ 0.454, *P* = .01) and MT staining (ꞇ 0.628, *P* = .01) of colonic biopsy specimens were also both moderately correlated with an outcome of death because of GI disease using Kendall's tau‐b.

All results from statistical analyses for the clinicopathologic variables for duodenal and colonic specimens are presented in Tables 3 and 4, respectively. Multivariable analysis was not carried out because of limited predictors identified in the univariable analysis (Table 3) and small sample size (Table 4). All results from statistical analysis for outcome variables are presented in Table 5.

**TABLE 3:** Univariable logistic regression analysis of clinicopathologic variables with the presence of fibrosis in 57 duodenal biopsy specimens of cats with chronic inflammatory enteropathy.

Variable	Number of cats in analysis	Masson's trichrome		Vimentin	
		OR (95% CI)	*P* value	OR (95% CI)	*P* value
Body weight (kg)	57	1.34 (0.83‐2.22)	.22	1.83 (1.05‐3.18)	**.03**
Body condition score out of 9	57	1.30 (0.88‐1.92)	.19	1.52 (1.02‐2.29)	**.04**
History of diarrhea	57	0.74 (0.26‐2.12)	.57	0.70 (0.23‐2.12)	.53
History of weight loss	57	0.47 (0.16‐1.40)	.18	0.68 (0.23‐2.04)	.49
Total protein (g/L)	54	1.02 (0.96‐1.10)	.46	1.02 (0.95‐1.09)	.56
Albumin (g/L)	54	1.09 (0.97‐1.21)	.15	1.08 (0.96‐1.21)	.21
Inorganic phosphorous (mmol/L)	51	1.28 (0.31‐5.30)	.74	2.07 (0.48‐8.97)	.33
Cobalamin (ng/L)	42	1.00 (0.99‐1.00)	.30	1.00 (0.99‐1.00)	.38
Alanine aminotransferase (U/L)	54	1.00 (0.98‐1.01)	.40	1.00 (0.98‐1.01)	.52
Alkaline phosphatase (U/L)	54	0.99 (0.98‐1.01)	.40	1.00 (0.98‐1.01)	.86
Thickening of intestinal muscularis propria layer on ultrasound (yes)	57	1.57 (0.54‐4.58)	.41	0.784 (0.26‐2.37)	.67
WSAVA score of duodenum	57	0.99 (0.85‐1.44)	.84	1.07 (0.91‐1.25)	.41

*Note*: Results are presented as odds ratio (OR) and 95% confidence intervals (CI).

Abbreviation: WSAVA, World Small Animal Veterinary Association.

Significant *P* values are listed in bold.

**TABLE 4:** Univariable logistic regression analysis of clinicopathologic variables with the presence of fibrosis in 29 colonic biopsy specimens of cats with chronic inflammatory enteropathy.

Variable	Number of cats in analysis	Masson's trichrome		Vimentin	
		OR (95% CI)	*P* value	OR (95% CI)	*P* value
Body weight (kg)	29	0.98 (0.56‐1.73)	.94	1.01 (0.54‐1.87)	.98
Body condition score (0‐9)	29	1.09 (0.67‐1.75)	.74	0.88 (0.51‐1.55)	.66
History of weight loss	29	0.30 (0.05‐1.86)	.20	1.30 (0.22‐7.87)	.78
Total protein (g/L)	29	0.96 (0.88‐1.06)	.44	0.98 (0.88‐1.08)	.67
Albumin (g/L)	29	1.01 (0.85‐1.21)	.91	1.01 (0.83‐1.23)	.96
Inorganic phosphorous (mmol/L)	29	0.39 (0.03‐5.98)	.50	0.04 (0.00‐2.67)	.13
Cobalamin (ng/L)	20	1.00 (1.00‐1.02)	.29	1.00 (1.00‐1.02)	.29
Alanine aminotransferase (U/L)	29	0.98 (0.94‐1.02)	.37	0.98 (0.93‐1.03)	.37
Alkaline phosphatase (U/L)	29	1.00 (0.97‐1.02)	.65	0.99 (0.96‐1.03)	.58
WSAVA score of colon	29	1.25 (0.94‐1.65)	.13	1.14 (0.86‐1.52)	.36

*Note*: Results are presented as odds ratio (OR) and 95% confidence intervals (CI).

Abbreviation: WSAVA, World Small Animal Veterinary Association.

**TABLE 5:** The association between the presence of intestinal mucosal fibrosis in the duodenum or colon of cats with chronic inflammatory enteropathy and outcome.

Biopsy specimen	Outcome	Masson's trichrome		Vimentin	
		Kendall's tau‐b	*P* value	Kendall's tau‐b	*P* value
Duodenal (n = 49)	Attainment of clinical remission	−0.130	.37	−0.243	.09
	Death because of gastrointestinal disease	0.097	.50	0.198	.17
Colonic (n = 23)	Attainment of clinical remission	−0.335	**.03**	−0.335	**.03**
	Death because of gastrointestinal disease	0.628	**.01**	0.454	**.01**

Significant *P* values are listed in bold.

### 3.7 Composite collagen scoring

Of the 20 cats that had duodenal specimens that underwent IHC for ACA, 11 cats (55%) were given a CCS between 0 and 1 to indicate mildly increased collagen, 7 cats (35%) were given a CCS between 2 and 4 to indicate moderately increased collagen and 2 cats (10%) were given a CCS of ≥6 to indicate markedly increased collagen. The scoring breakdowns are depicted in Table 6.

**TABLE 6:** Composite collagen score combinations and number of cats with this scoring combination.

Collagen intensity × collagen frequency	Composite collagen score	Number of cats with this scoring combination
0 × 0	0	1
1 × 0	0	5
1 × 1	1	5
1 × 2	2	2
2 × 1	2	3
2 × 2	4	2
3 × 2	6	2

*Note*: A collagen frequency score of 0 referred to a visual estimate of area labeled as <25%.

### 3.8 Results of association analyses for IHC of collagen I

Kendall's tau‐c correlation of CCS indicated that a history of weight loss was strongly correlated with increased CCS (ꞇ 0.630, *P* < .001). Spearman's rank correlation indicated that decreased serum albumin concentration (*r*_s_ −.179, *P* < .001) was weakly correlated with CCS. Spearman's rank correlation indicated that decreased serum cobalamin concentration (*r*_s_ −.740, *P* = .01) was strongly correlated with CCS. Additionally, increased WSAVA score of the duodenum was moderately correlated with CCS (*r*_s_ .554, *P* = .01). All remaining results from statistical analyses are presented in Table 7.

**TABLE 7:** Spearman's rank correlation and Kendall's tau c* correlation coefficient were used to assess the correlation between clinicopathologic variables and composite collagen score on 20 duodenal biopsy specimens of cats with chronic inflammatory enteropathy and increased duodenal mucosal fibrosis.

Variable	Number of cats in analysis	Correlation co‐efficient	*P* value
Body weight (kg)	20	0.337	.50
Body condition score out of 9	20	−0.144	.55
History of diarrhea	20	−0.200*	.41
History of weight loss	20	0.630*	**<.001**
Total protein (g/L)	19	−0.279	.25
Albumin (g/L)	19	−0.719	**<.001**
Inorganic phosphorous (mmol/L)	19	−0.119	.63
Cobalamin (ng/L)	12	−0.740	**.01**
Alanine aminotransferase (U/L)	20	−0.320	.17
Alkaline phosphatase (U/L)	20	−0.143	.55
Thickening of intestinal muscularis propria layer on ultrasound	20	−0.080*	.75
WSAVA score of duodenum	20	0.554	**.01**
Attainment of clinical remission	20	−0.110*	.64
Outcome of death because of gastrointestinal disease	20	−0.280*	.24

Abbreviation: WSAVA, World Small Animal Veterinary Association.

Significant *P* values are listed in bold.

## 4 DISCUSSION

We identified duodenal and colonic mucosal fibrosis as a common finding in cats with CIE. When using HE staining alone, IF was identified in 51% (n = 29) and 76% (n = 22) of duodenal and colonic biopsy samples respectively. However, use of MT stain allowed for identification of IF in 58% (n = 33) of duodenal biopsy specimens. Additionally, immunolabeling of vimentin allowed for identification of IF in 65% (n = 37) and 79% (n = 23) of duodenal and colonic biopsy specimens, respectively. Additionally, we found that various clinicopathological variables, as well as outcome, were correlated with the presence of IF.

The presence of mucosal fibrosis, as detected by positive vimentin labeling of duodenal biopsy specimens, was correlated with increased BW and BCS. Evidence in studies of humans with chronic obesity have identified body mass index as a predictive marker for active IBD.^22^, ^23^, ^24^ Additionally, increased mesenteric fat around intestinal segments, so‐called “creeping fat” as seen in Crohn's disease, also has been directly linked to increased intestinal wall fibrosis in humans with IBD.^25^ No such parallel has been identified in cats, and a recent study evaluating disease associations in 9062 overweight and obese cats found no correlation between increased BW or BCS and GI disease.^26^ Additional investigation of these variables, and consideration of the use of percentage weight change as a more discriminating measure, are required to better understand the relationships among BW, BCS, and IF in cats with CIE.

A history of diarrhea was weakly but positively correlated with the presence of IF as identified by MT staining of colonic biopsy specimens. In humans with IBD, diarrhea is thought to lead to a self‐perpetuating cycle by instigating a state of chronic inflammation that propagates the development of IF, which in turn further exacerbates diarrhea because of impaired intestinal wall function.^27^, ^28^ The severity of diarrhea in humans with IBD is considered an important determinant of clinical disease activity.^29^ This conclusion is similar to the Feline Chronic Enteropathy Activity Index, which correlated the presence of frequent diarrhea with increased histologically identified intestinal inflammation.^30^ Our study only assessed history of diarrhea as a binary variable (yes vs no). Therefore, determining diarrhea severity and characteristics (i.e., small, mixed, or large bowel) could more specifically correlate diarrhea with the histologic presence of IF. Additionally, it would help to examine the value of obtaining IBSp in all cats that are presented with diarrhea. Future studies also should evaluate the potential effect of IF on colonic motility.

Thickening of the intestinal muscularis propria layer on transabdominal ultrasound examination was correlated with the presence of colonic mucosal fibrosis as detected by IHC labeling for vimentin. In humans with IBD, increased colonic wall thickness, as recognized ultrasonographically, has been positively correlated with histologic inflammation and IF.^31^ One study that evaluated ultrasonographically‐identified SI muscularis propria thickening in cats identified low positive predictive value of 18.9% to 57.1% for histologic disease.^32^ Although the aforementioned study did not examine the colon, its results potentially could be extrapolated similarly. However, an additional consideration is that ultrasonographic studies in clinically healthy cats have repeatedly identified the colon to be the thinnest segment of the GI tract.^33^, ^34^ Therefore, it also could be hypothesized that colonic muscularis propria thickening identified in cats with GI signs and CIE is clinically relevant and should not be disregarded. Along with our results, these findings should prompt the specific examination of the relationship between colonic ultrasonography and IF, which has not been assessed previously. Additionally, further evaluation of vimentin as a sensitive marker for IF is warranted.

The use of ACA resulted in a moderate correlation between CCS and composite WSAVA duodenal score, which is likely a reflection of the unblinded process by which the 20 cases were selected. This correlation therefore could emphasize how individual WSAVA scoring variables are more reflective of the intricacies between clinicopathologic and histopathologic relationships than the composite score. This conclusion is supported by 1 study in which measuring colonic fibrosis as an individual score resulted in a better correlation with clinical activity in dogs with immunosuppressant‐responsive enteropathy.^35^ Additionally, because fibrosis occurs as a sequela to inflammation, it is possible that the presence of inflammatory cells and fibrosis are inverse to each other, which may not be accounted for in the composite WSAVA score.

A history of weight loss and decreased serum cobalamin concentration were strongly correlated with the CCS with decreased serum albumin concentration demonstrating a weak correlation. Hypocobalaminemia and hypoalbuminemia previously have been recognized in cats with chronic GI disease, but no dependent relationship has been described between the 2 or reported in relation to IF.^36^, ^37^, ^38^ In humans, IBD is characterized by marked GI inflammation, which causes excessive enterocyte turnover and IF.^9^, ^39^ This situation subsequently causes loss of the intestinal barrier function and macronutrient and micronutrient deficiency, which could similarly occur in cats with CIE.^40^ The relationship identified between hypocobalaminemia and duodenal mucosal collagen I deposition also could represent the diffuse nature of IF throughout the GI tract, given that cobalamin is absorbed in the distal ileum.^36^ However, it also could point to a distinct relationship between IF and SI dysbiosis with growing evidence linking disruptions in intestinal microbiota to intestinal fibrogenesis in humans with IBD.^41^ Studies in cats with CE have identified the presence of dysbiosis in 76% of affected cats.^42^ Furthermore, another study identified increased numbers of mucosa‐associated Enterobacteriaceae in cats with duodenal CIE, however, this finding did not correspond to increased duodenal mucosal fibrosis.^43^

Positive vimentin labeling and MT staining of colonic biopsy specimens were positively correlated with failure to attain clinical remission and increased likelihood of death because of GI disease. Possible reasons for this observation are that cats that enter clinical remission have less or no IF or that, in the event of CIE relapse, the presence of IF results in difficulty in re‐gaining clinical remission. A recent study identified collagen I in later‐stage intestinal fibrosis relative to collagen III.^12^ Therefore, diagnostic delay in identifying IF could further correlate advanced collagen deposition with failure to attain clinical remission. However, additional studies evaluating the role of collagen III in IF are required to better understand this relationship.

Interestingly, we observed failure to attain clinical remission and increased likelihood of death because of GI disease with the presence of IF in colonic specimens only. In humans with IBD, proinflammatory and profibrotic cytokines are upregulated in the colon,^44^, ^45^ which could occur similarly in cats with CIE. Because luminal bacterial load is higher in the colon compared to the small intestine,^46^ dysregulation and mucosal fibrosis may be propagated by this more pronounced, and microbially‐mediated, inflammatory milieu. One study identified that mucosa‐adherent *Fusobacterium* spp. were increased in colonic biopsy specimens of cats with small cell lymphoma (SCL) relative to cats with CIE.^47^ Further understanding of these particular bacterial alterations could help identify whether certain species of bacteria facilitate CIE and fibrogenesis. Our results emphasize the value of colonic sampling in the assessment of CIE in cats, which may be greater than has been previously appreciated.

The associations among IF, clinical remission, and death caused by CIE are likely intrinsically related. Consequently, our results provide an alternative perspective on future management of cats with CIE by specifically aiming to minimize intestinal fibrogenesis. Inhibition of IF is an already established therapeutic target in humans with IBD.^15^, ^16^, ^31^, ^48^ Biological medicines to antagonize profibrotic cytokines and gut‐selective integrin, which selectively inhibits the infiltration of leukocytes into the GI submucosa, are some treatment options.^28^, ^49^, ^50^, ^51^ Studies are needed to assess these medications in cats and in CIE. Our results indicate that alternative treatment options, yet to be explored, potentially could correlate with better outcome in cats with CIE if IF is present. Additionally, our results emphasize the importance of additional stains for improved identification of IF at the time of histological diagnosis of CIE in cats because of their influence on forecasting prognosis.

Our study had some limitations. The retrospective study design resulted in a small sample size. Multiple clinicians were involved in diagnostic investigations and in performing ultrasound examinations which could have led to increased interobserver variability^52^ and variability in the procurement of biopsy specimens. Additionally, not all cats had feline leukemia virus and feline immunodeficiency virus testing to exclude these infectious diseases. In humans with IBD, intestinal fibrosis can be a feature in subclinical disease.^5^ The identification of subclinical CIE in cats remains challenging. However, mucosal fibrosis has been identified in the IBSp of clinically healthy cats.^53^ Additional longitudinal investigations could investigate whether the presence of intestinal mucosal fibrosis in clinically healthy cats could predict future clinical CIE. Univariable analysis was only available for association analysis and results obtained from statistical analyses differed between stains, which could suggest possible type I error. Post hoc comparison tests and adjusted *P* values would be beneficial for future analyses investigating a larger number of cats. Quantification of the frequency of collagen I immunolabeling could have been assessed using image processing programs such as ImageJ.^54^ However, we believed that visual estimation by the pathologist would be more representative of how IBSp generally are evaluated in clinical practice, and also allowed for the detection of subtle changes in tissue architecture that could be missed using digital techniques. Biopsy specimens examined were those already collected at the time of diagnosis, not allowing for broader assessment of the GI tract. Consistent with this concern, ileal biopsies were not consistently performed, which could have resulted in missed intestinal neoplasia, considering that SCL is most common in the ileum and jejunum.^1^, ^55^ However, evaluation of duodenal and colonic biopsy specimens in our study is representative of how intestinal specimens commonly are obtained in clinical practice.^56^ Additionally, despite a study suggesting that incorporation of IHC and PCR for antigen receptor rearrangements increases the ability of SCL identification in IBSp from cats, it also found that ileal specimens seldom changed the diagnosis achieved from duodenal specimens alone.^57^

## 5 CONCLUSIONS

Our results identified that IF is a frequent finding in cats with CIE. Additionally, we found that use of MT staining and immunolabeling for vimentin allowed for better identification of IF in IBSp relative to routine HE staining.

Our study is the first to describe a relationship between IF and outcome in cats with CIE. The presence of colonic mucosal fibrosis, identified using MT staining and IHC for vimentin, was correlated with failure to attain clinical remission and death because of GI disease. This information is clinically relevant in anticipating the course of CIE in cats as a relapsing‐remitting disease. Our results can serve as a stimulus for further research into the complex role of IF in CIE in cats and into IF as a potential treatment target.

## CONFLICT OF INTEREST DECLARATION

Authors declare no conflict of interest.

## OFF‐LABEL ANTIMICROBIAL DECLARATION

Authors declare no off‐label use of antimicrobials.

## INSTITUTIONAL ANIMAL CARE AND USE COMMITTEE (IACUC) OR OTHER APPROVAL DECLARATION

Approved by the Clinical Research Ethical Review Board at the Royal Veterinary College (RVC), URN 2020 1993‐3.

## HUMAN ETHICS APPROVAL DECLARATION

Authors declare human ethics approval was not needed for this study.
